# Epigenetic silencing of a long non-coding RNA *KIAA0495* in multiple myeloma

**DOI:** 10.1186/s12943-015-0444-8

**Published:** 2015-09-26

**Authors:** Kwan Yeung Wong, Zhenhai Li, Xiaoqin Zhang, Gilberto Ka Kit Leung, Godfrey Chi-fung Chan, Chor Sang Chim

**Affiliations:** Department of Medicine, Queen Mary Hospital, The University of Hong Kong, Pokfulam Road, Pokfulam, Hong Kong; Department of Surgery, Queen Mary Hospital, The University of Hong Kong, Pokfulam Road, Pokfulam, Hong Kong; Department of Paediatrics and Adolescent Medicine, Queen Mary Hospital, The University of Hong Kong, Pokfulam Road, Pokfulam, Hong Kong

**Keywords:** Long non-coding RNA, *KIAA0495*, DNA methylation, Myeloma, Tumour suppressor

## Abstract

In multiple myeloma, a long non-coding RNA, *KIAA0495* (alias *PDAM*/*TP73-AS1*), had been found progressively downregulated from normal plasma cell to benign monoclonal gammopathy of undetermined significance to symptomatic myeloma. Herein, by methylation-specific PCR, the putative *KIAA0495* promoter was found unmethylated in all healthy controls (*N* = 14) but methylated in 50 % of myeloma cell lines (*N* = 10). *KIAA0495* methylation was shown inversely correlated with *KIAA0495* expression. However, *KIAA0495* methylation was detected in none of both primary myeloma samples at diagnosis (*N* = 61) and at relapse/progression (*N* = 16). Collectively, despite frequently methylated in cell lines, *KIAA0495* methylation appeared unimportant in the pathogenesis or progression of myeloma.

**Electronic supplementary material:**

The online version of this article (doi:10.1186/s12943-015-0444-8) contains supplementary material, which is available to authorized users.

## Introduction

Long non-coding RNA (lncRNA) is a novel class of functional RNA molecules >200 nucleotides with little or no protein-coding capacity [1, 2]. Based on their genomic locations relative to annotated protein-coding genes, lncRNAs are broadly classified into intergenic, intragenic, antisense, pseudogene, or divergent transcripts [3, 4]. Functionally, lncRNAs play a role in, but not limited to, development, differentiation, and carcinogenesis [2, 5]. Moreover, lncRNAs are found dysregulated and therefore potentially to be oncogenic or tumour suppressive in human cancers [6, 7].

**Table 1:** Primer sequences and PCR reaction conditions

Primer set	Forward primer (5’ – 3’)	Reverse primer (5’ – 3’)	Product size (bp)	MgCl_2_/Tm/cycles	Reference
(I) Methylation-specific polymerase chain reaction (MSP)					
*KIAA0495*					
M-MSP	TGG AGA TAA CGG GTT TAG AGA AAT C	AAC GAA AAC AAA AAT AAA ACC TTC G	191	1.5 mM/58 °C/40X	--
U-MSP	TGG AGA TAA TGG GTT TAG AGA AAT T	AAA CAA AAA CAA AAA TAA AAC CTT CA	192	2.0 mM/58 °C/40X	--
(II) Reverse transcription-polymerase chain reaction (RT-PCR)					
*KIAA0495*	GCT GCT TGC TGT ACG TGG TG	CGT GGC TGA CAC AAA CTT GC	178	1.5 mM/60 °C/35X	[16]
*GAPDH*	ACC ACA GTC CAT GCC ATC ACT	TCC ACC ACC CTG TTG CTG TA	452	1.5 mM/60 °C/24X	

Keys: Tm, annealing temperature; M-MSP, MSP for the methylated allele; U-MSP, MSP for the unmethylated allele

Multiple myeloma is a form of haematological cancer originated from malignant transformation of plasma cells [8]. Clinically, this disease begins with benign monoclonal gammopathy of undetermined significance (MGUS), some of which may undergo asymptomatic smouldering multiple myeloma (SMM), and progresses to symptomatic myeloma [9]. Genetically, despite D-type cyclins are apparently upregulated in all patients, myeloma is a heterogeneous disease characterized by specific gains or losses of chromosomes, or reciprocal translocations, such as t(4;14)(p16.3;q32) and t(14;16)(q32;q23) [10].

DNA methylation refers to the catalytic addition of a methyl (−CH_3_) group to the cytosine ring of a CpG dinucleotide [11]. Human cancers are characterized by loss of global DNA methylation but gain of methylation at promoter-associated CpG islands (a cluster of CpG dinucleotides) and hence transcriptional silencing of specific tumour suppressor genes or miRNAs [12]. In myeloma, DNA methylation has been shown to mediate silencing of multiple tumour suppressor genes and miRNAs, and has been implicated in the pathogenesis and prognosis of the disease [13, 14].

Interestingly, by gene expression profiling, *KIAA0495*, a lncRNA transcribed from chromosome 1p36, has been shown to be progressively downregulated from normal plasma cell to MGUS to symptomatic myeloma [15]. Moreover, methylation-mediated silencing of *KIAA0495* has been demonstrated in oligodendroglial tumours, leading to enhanced cisplatin resistance via upregulation of anti-apoptotic B-cell CLL/lymphoma 2-like 1 (BCL2L1) [16]. Hence, we hypothesized that DNA methylation may account for the progressive downregulation of *KIAA0495* in the pathogenesis of myeloma. Herein, we have studied and reported methylation of *KIAA0495* in myeloma cell lines, primary myeloma marrow samples at diagnosis, and at relapse/progression. Materials and methods have been incorporated as Additional file 1.

## Findings

### Methylation-specific PCR: *KIAA0495* methylation in healthy controls and myeloma cell lines

**Fig. 1:**
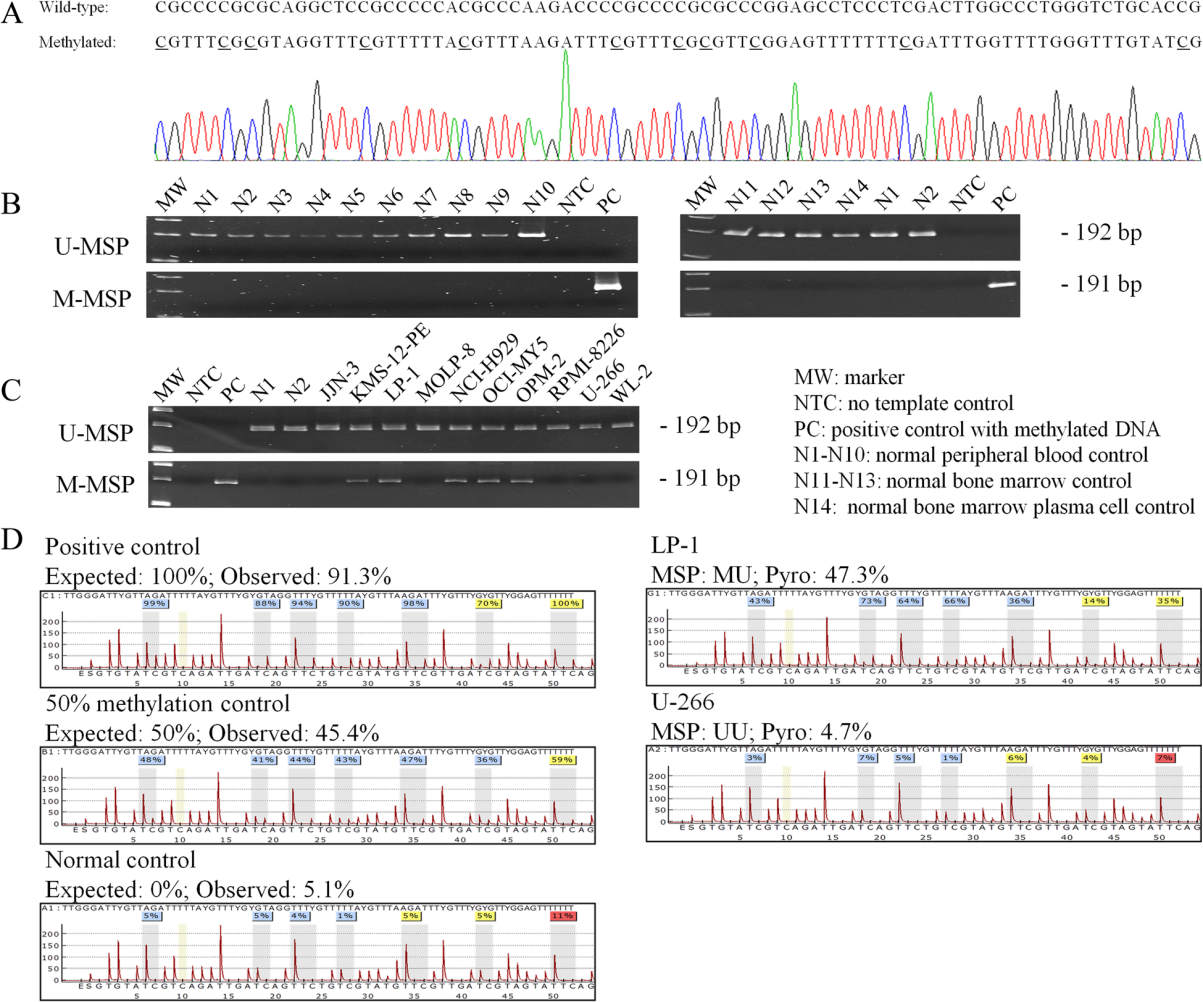
Methylation of *KIAA0495* in myeloma. **a** Sequencing analysis of M-MSP products showed that the cytosine [C] residues of CpG dinucleotides were methylated and remained unchanged after bisulfite conversion, whereas all the other non CpG C residues were unmethylated and converted to thymidine [T], indicating complete bisulfite conversion and specificity of MSP. **b** M-/U-MSP showed *KIAA0495* was completely methylated in positive control with methylated DNA, but completely unmethylated in 14 healthy donor controls, including peripheral buffy coat (N1-N10), marrow buffy coat (N11-N13), and CD138-sorted marrow plasma cells (N14). **c** M-/U-MSP showed *KIAA0495* was partially methylated in KMS-12-PE, LP-1, NCI-H929, OCI-MY5, and OPM-2, whereas it was completely unmethylated in MOLP-8, RPMI-8226, U-266, WL-2, and JJN-3. **d** Quantitative bisulfite pyrosequencing interrogating the methylation intensity on a stretch of 7 neighboring CpG dinucleotides indicated consistent resutls as shown by the MSP statuses (MM, MU, and UU)

While the putative *KIAA0495* promoter region was found embedded in a CpG island (Additional file 2: Figure S1), MSP primers were designed to study methylation of this CpG island in a panel of healthy controls [peripheral (*N* = 10) and marrow (*N* = 3) buffy coat, CD138-sorted healthy plasma cell (*N* = 1)], and myeloma cell lines (*N* = 10) (Table 1). Direct sequencing analysis of M-MSP products from bisulfite-treated positive control showed expected conversion of unmethylated cytosine into uracil (turned to thymidine after PCR), with methylated cytosine remained unchanged, indicating complete bisulfite conversion and MSP specificity (Fig. 1a). None of 14 healthy controls showed *KIAA0495* methylation (Fig. 1b). On the other hand, in myeloma cell lines, KMS-12-PE, LP-1, NCI-H929, OPM-2, and OCI-MY5 were partially methylated, whereas MOLP-8, RPMI-8226, U-266, WL-2, and JJN-3 were completely unmethylated for *KIAA0495* (Fig. 1c). Moreover, the MSP methylation statuses of controls and cell lines were confirmed by quantitative bisulfite pyrosequencing (Fig. 1d). These data suggested that methylation of *KIAA0495* was tumour-specific, consistent with other tumour suppressor protein-coding genes and non-coding miRNAs in myeloma [17–20].

### *KIAA0495* methylation and expression in myeloma cell lines

**Fig. 2:**
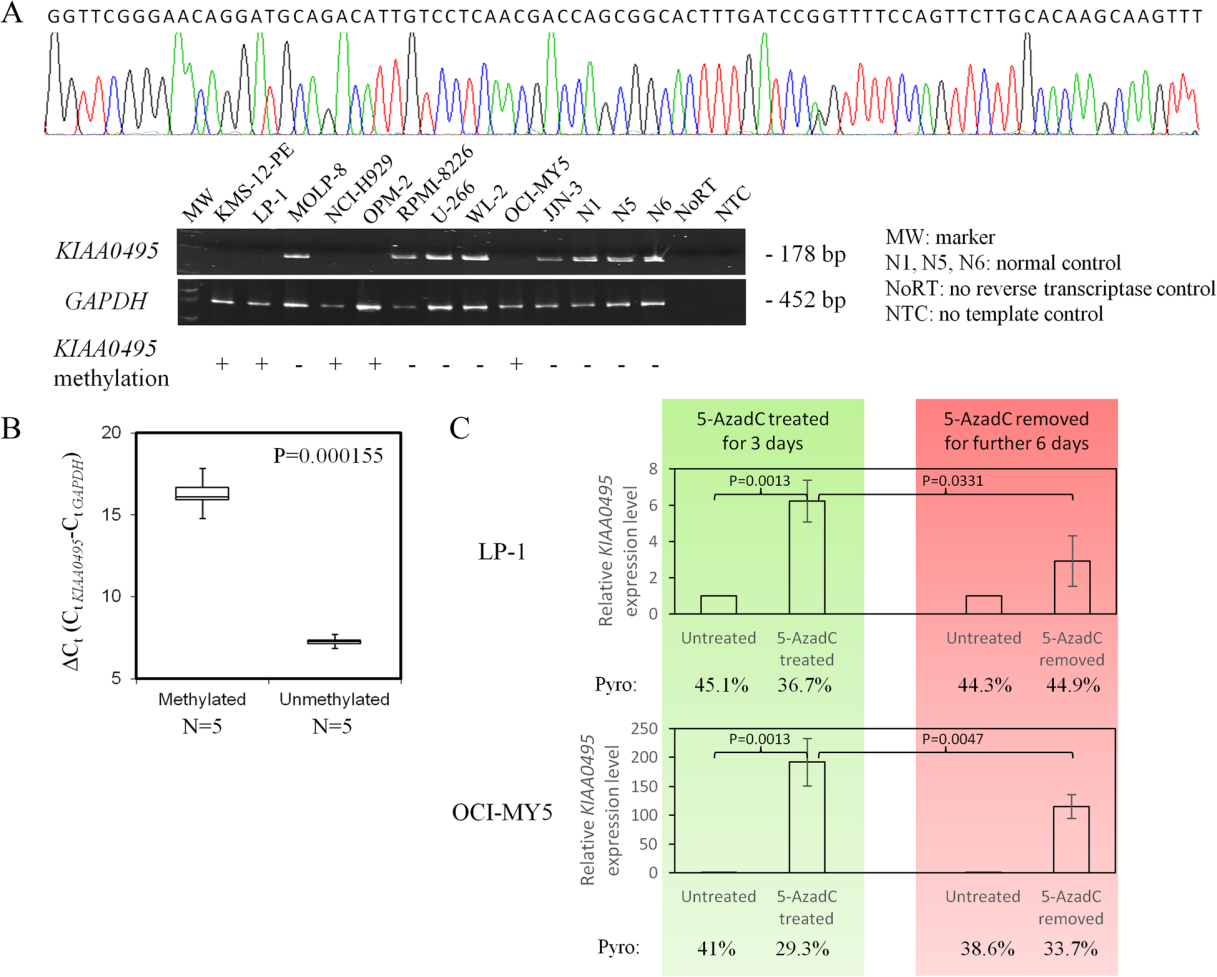
Methylation and expression of *KIAA0495* in myeloma cells. **a** Semi-quantitative RT-PCR analysis showed expression of *KIAA0495* in MOLP-8, RPMI-8226, U-266, WL-2, and JJN-3, which were completely unmethylated for *KIAA0495*, but not in KMS-12-PE, LP-1, NCI-H929, OCI-MY5, and OPM-2, which were partially methylated for *KIAA0495* (*P* = 0.0079). Sequencing analysis confirmed the authenticity of the RT-PCR of *KIAA0495*. **b** qRT-PCR analysis showed the expression of *KIAA0495* was significantly higher, as indicated by a lower ΔC_t_, in methylated than completely unmethylated myeloma cell lines (*P* = 0.000155). **c** Two partially methylated myeloma cell lines, LP-1 and OCI-MY5, were separately treated with 1 μmol/l of 5-AzadC for 3 days, after which, 5-AzadC was removed and cells were cultured for additional 6 days. On day 3, upon treatment with 5-AzadC, *KIAA0495* was demethylated as indicated by the decreased percentage of methylation intensity by quantitative bisulfite pyrosequencing, with concommitent *KIAA0495* re-expression as measured by qRT-PCR. On day 9, upon removal of 5-AzadC for 6 days, methylation of *KIAA0495* was restored with re-suppression of *KIAA0495*

To determine whether *KIAA0495* methylation was associated with silencing of *KIAA0495* expression, the expression level of *KIAA0495* was measured by semi-quantitative RT-PCR and quantitative real-time PCR (qPCR), and correlated with the *KIAA0495* methylation status as detected by MSP in myeloma cell lines. Of the 10 myeloma cell lines, *KIAA0495* methylation was associated with lower expression as detected by both semi-quantitative RT-PCR (*P* = 0.0079; Fig. 2a) and quantitative real-time PCR (*P* = 0. 000155; Fig. 2b).

Moreover, myeloma cell lines with *KIAA0495* methylation were treated with a hypomethylating agent, 5-AzadC, followed by pyrosequencing and qPCR analyses. Upon 5-AzadC treatment, LP-1 and OCI-MY5 cells, which were partially methylated for *KIAA0495*, showed progressive demethylation of the *KIAA0495* promoter, as evidenced by the decreased methylation percentage on a stretch of seven consecutive CpG dinucleotides, together with concomitant re-expression of the *KIAA0495* transcript, as illustrated by the increased expression relative to the untreated control (LP-1: *P* = 0.0013; OCI-MY5: *P* = 0.0013; Fig. 2c). Nevertheless, when 5-AzadC-treated cells were continually cultured with fresh medium in the absence of 5-AzadC for a further 6 days, methylation of the *KIAA0495* promoter was restored, with simultaneous suppression of the *KIAA0495* expression (LP-1: *P* = 0.0331; OCI-MY5: *P* = 0.0447; Fig. 2c). Taken together, the methylation of *KIAA0495* was inversely correlated with *KIAA0495* expression level in myeloma cell lines, similar to the methylation-mediated silencing of *KIAA0495* demonstrated in glioma cell lines and primary oligodendroglial tumour cells [16], suggesting that methylation of the promoter-associated CpG island emerged to be one of the mechanisms resulting in the regulation of lncRNAs in cancer cells.

### Methylation-specific PCR: *KIAA0495* methylation in myeloma primary samples at diagnosis and at relapse

**Fig. 3:**
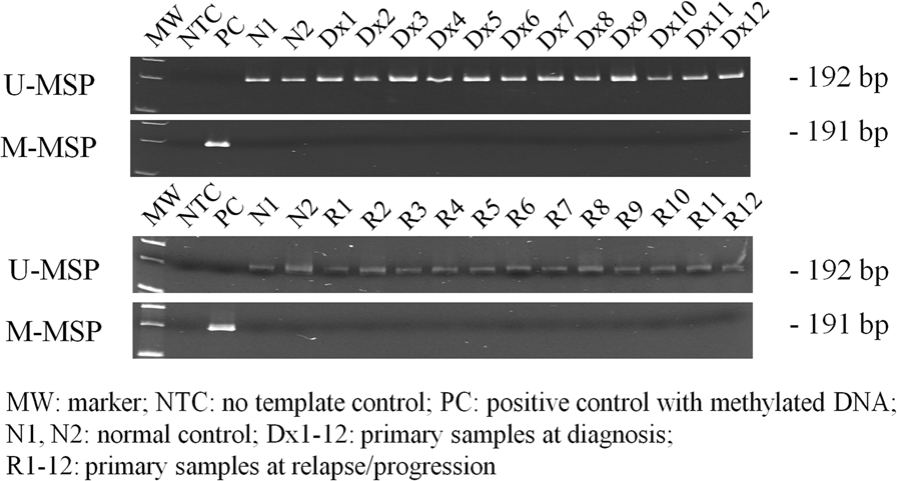
Methylation of *KIAA0495* in primary myeloma marrow samples at diagnosis (Dx) and at relapse/progression (R)

To examine if methylation of *KIAA0495* was also detected in primary samples, methylation of *KIAA0495* was studied in 61 primary samples at diagnosis and 16 primary samples at relapse by MSP. However, none of these samples showed methylation of *KIAA0495* (Fig. 3), indicating methylation of *KIAA0495* was rarely detected in primary myeloma samples. Hence, similar to the studies of *miR-9-1*, *miR-9-3*, and *miR-124-1* [21, 22], these data suggested that methylation of certain tumour suppressor non-coding miRNAs or lncRNAs was acquired *in vitro* during continuous culture of myeloma cells, hence not pathogenic. Therefore, methylation was not the mechanism leading to the progressive downregulation of *KIAA0495* from normal plasma cell to MGUS to symptomatic myeloma [15], suggestive of other mechanisms, such as histone modification and miRNA-mediated repression, may come into play. For instance, in gastric cancer cells, histone deacetylation was found to be associated in the downregulation of a tumour suppressor lncRNA, *FENDRR* [23]. Moreover, in breast cancer cells, miR-21 was shown to target and hence downregulate the expression of a tumour suppressor lncRNA, *GAS5* [24]. Furthermore, while *KIAA0495* resides on 1p36, which has been shown frequently deleted in newly diagnosed patients with myeloma [25], the loss of expression of *KIAA0495* may be associated with chromosome deletion. Last but not least, inactivation of *KIAA0495* may also be accounted by haploinsufficiency, which has been demonstrated in the *TP53* in myeloma [26].

## Conclusion

Methylation of *KIAA0495* is tumour-specific, leading to reversible silencing of *KIAA0495* expression in myeloma cell lines. On the other hand, it is rarely detected in primary samples of myeloma at diagnosis or at relapse, and hence not responsible for the progressive downregulation of *KIAA0495* from normal plasma cell to MGUS to symptomatic myeloma, as shown by the gene expression profiling, and remains as a possible *in vitro* event acquired during continuous culture of myeloma cells.

## Additional files

Additional file 1:
**Materials and methods.** (DOCX 20 kb) Additional file 2: Figure S1. Schematic diagram showing the transcription start sites (TSS) of *KIAA0495*, distribution of CpG dinucleotides (red lines), and amplicons of methylation-specific PCR (green box) and pyrosequencing (purple box). (TIFF 339 kb)

## Abbreviations

lncRNA: Long non-coding RNA
MGUS: Monoclonal gammopathy of undetermined significance
miRNA: microRNA
MSP: Methylation-specific PCR
M-MSP: Methylated MSP
U-MSP: Unmethylated MSP
PCR: Polymerase chain reaction
RT-PCR: Reverse transcription-PCR
SMM: Smouldering multiple myeloma
