# Supplementary material for: Epigenetic silencing of a long non-coding RNA KIAA0495 in multiple myeloma
Source: Mol Cancer. 2015 Sep 26;14:175. doi: 10.1186/s12943-015-0444-8 (PMC4583761; doi:10.1186/s12943-015-0444-8)
Supplement: Additional file 1: — Materials and methods. (DOCX 20 kb) [file 12943_2015_444_MOESM1_ESM.docx]

**Materials and methods**

Patient samples

Bone marrow samples were obtained from 61 myeloma patients at diagnosis and 16 myeloma patients at relapse/progression. The diagnosis of myeloma was based on standard criteria of International Myeloma Working Group [[1](#_ENREF_1)]. Complete staging work-up included bone marrow examination, skeletal survey, serum and urine protein electrophoresis, and serum immunoglobulin levels. Among the 61 newly diagnosed myeloma cases, there were 27 females and 34 males, with a median age of 67 (35-91) years. Apart from 12 patients with insufficient clinical data of International Staging System (ISS) [[2](#_ENREF_2)], there were 9 stage I, 25 stage II, and 15 stage III cases. Based on immunoglobin isotype data, there were 14 IgA, 41 IgG, 5 light chain, and 1 non-secretary cases. According to the criteria of the European Group for Blood and Marrow Transplantation Myeloma Registry [[3](#_ENREF_3)], “relapse” from complete remission (CR) was defined as the reappearance of the same paraprotein detected by serum/urine protein electrophoresis, appearance of new bone lesion or extramedullary plasmacytoma, or unexplained hypercalcaemia. The study has been approved by Institutional Review Board of Queen Mary Hospital, and written informed consent was obtained from patient for publication of this report and any accompanying data or images.

Cell culture

Human myeloma cell lines LP-1 and RPMI-8226 were kindly provided by Dr. Robert Orlowski (Department of Lymphoma/Myeloma, Division of Cancer Medicine, The University of Texas MD Anderson Cancer Center, Houston, TX, USA), JJN-3 and OCI-MY5 by Dr. Wee Joo Chng (Department of Medicine, Yong Loo Lin School of Medicine, National University of Singapore), and WL-2 by Prof. Andrew Zannettino (Myeloma Research Programme, The University of Adelaide, Australia). NCI-H929 was purchased from American Type Culture Collection (Manassas, VA, USA). Other myeloma cell lines (KMS-12-PE, MOLP-8, OPM-2 and U-266) were purchased from Deutsche Sammlung von Mikroorganismen und Zellkulturen (DSMZ) (Braunschweig, Germany). Cells were cultured in RPMI-1640 medium (IMDM for LP-1), supplemented with 10% fetal bovine serum, 50 U/ml of penicillin and 50 ug/ml streptomycin, and incubated in a humidified atmosphere of 5% CO_2_ at 37°C. All cell culture reagents were purchased from Invitrogen (Carlsbad, CA, USA).

Methylation-specific polymerase chain reaction (MSP)

Genomic DNA was isolated from healthy donors [10 peripheral blood and 3 marrow buffy coat, and 1 CD138-sorted marrow plasma cells (AllCells, CA, USA)], primary myeloma marrow samples at diagnosis or at relapse/progression, and 10 myeloma cell lines (JJN-3, KMS-12-PE, LP-1, MOLP-8, NCI-H929, OCI-MY5, OPM-2, RPMI-8226, U-266 and WL-2) using QIAamp DNA Blood Mini Kit (Qiagen, Hilden, Germany). Bisulfite treatment of DNA for conversion of unmethylated cytosine to uracil was performed using EpiTect Bisulfite Kit (Qiagen, Hilden, Germany). Bisulfite-treated DNA was used as templates for methylated-MSP (M-MSP) and unmethylated-MSP (U-MSP) of KIAA0495. Primer sequences and conditions were listed in Table 1. Enzymatically methylated control DNA (Chemicon, Temecula, CA, USA), which would be positive for M-MSP and negative for U-MSP), was used as control in all MSP.

Quantitative bisulfite pyrosequencing

Bisulfite-treated DNA was used as template and amplified with a pair of methylation-unbiased primers, resulting a PCR product overlapping with the amplicon of the MSP. Forward primer: 5’- CAG GGA AAT CCT GTG GGG AGA G -3’; reverse primer: 5’- CCC ATC TAG GGA TCC ACA CC-3’; condition: 2mM/60°C/50X. A stretch of DNA with 9–12 adjacent CpG dinucleotides was pyrosequenced with sequencing primer: 5’- TGG GTG CAG GTG CAT - 3’.

Quantification of KIAA0495

Total RNA isolation was performed using mirVana™ miRNA Isolation Kit (Ambion, Austin, TX, USA), followed by reverse transcription to cDNA using QuantiTect Reverse Transcription Kit (Qiagen), according to the manufacturers’ instructions. The total cDNA was used as template to analyze KIAA0495 expression level in myeloma cell lines by semi-quantitative RT-PCR and quantitative RT-PCR (qRT-PCR) (iQ SYBR Green Supermix, Bio-Rad), with GAPDH as endogenous control. Specific primers of KIAA0495 were designed to span exons 1 and 2. Primer sequences and PCR conditions were listed in Table 1. Correlation between KIAA0495 methylation and expression detected by semi-quantitative RT-PCR and quantitative RT-PCR was studied by Chi-square test and Student’s t-test respectively.

5-Aza-2’-deoxycytidine (5-AzadC) treatment

LP-1 and OCI-MY5 cells, which were partially methylated for KIAA0495, were individually seeded in 25cm^2^ flasks at a density of 1×10^6^cells/ml. Cells were treated with 1 μmol/l of fresh 5-AzadC in every 24 hours for 3 days. Subsequantly, cells were allowed to grow in the absence of 5-AzadC for further 6 days. Cells on day 3 and 9 were harvested for DNA and RNA.

**References**

1. International Myeloma Working Group. **Criteria for the classification of monoclonal gammopathies, multiple myeloma and related disorders: a report of the International Myeloma Working Group**. *Br J Haematol*. 2003;**121**:749-57.

2. Greipp PR, Miguel JS, Durie BGM, Crowley JJ, Barlogie B, Blade J et al. **International Staging System for Multiple Myeloma**. *J Clin Oncol*. 2005;**23**:3412-20.

3. Blade J SD, Reece D, et al. **Criteria for evaluating disease response and progression in patients with multiple myeloma treated by high-dose therapy and haemopoietic stem cell transplantation**. *British Journal of Haematology*. 1998;**102**:1115-23.
