# Supplementary figures and images for: Epigenetic silencing of a long non-coding RNA KIAA0495 in multiple myeloma
Source: Mol Cancer. 2015 Sep 26;14:175. doi: 10.1186/s12943-015-0444-8 (PMC4583761; doi:10.1186/s12943-015-0444-8)

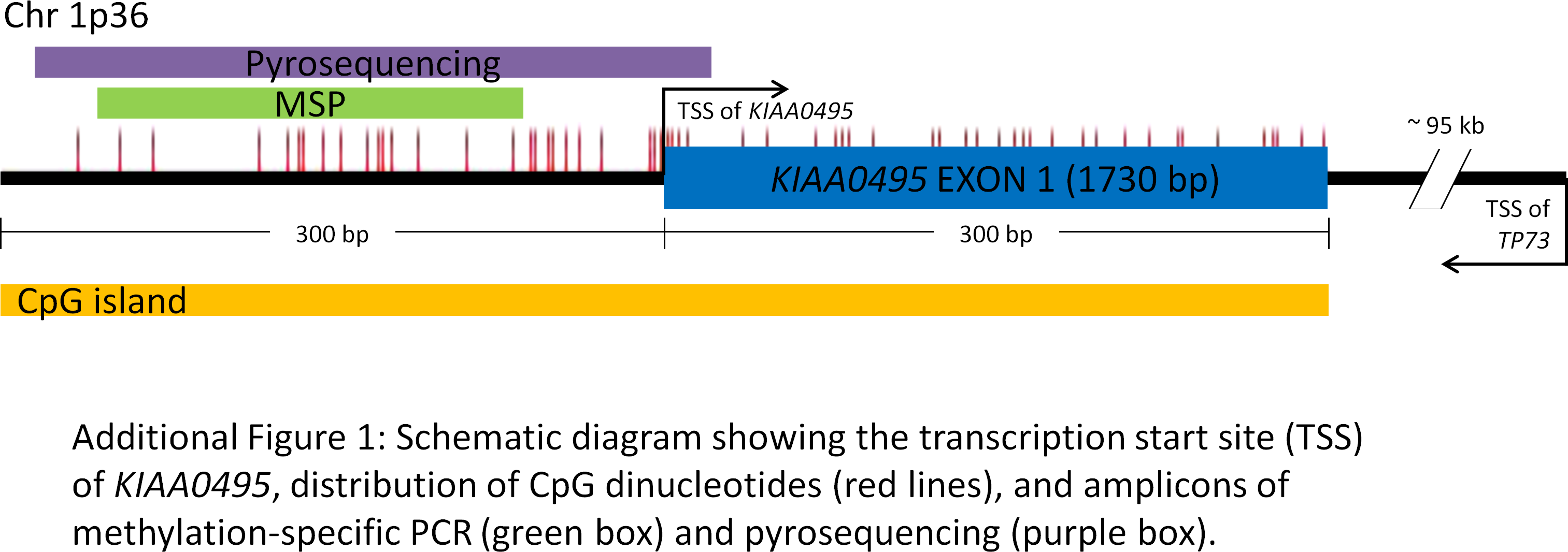

Supplement: Additional file 2: Figure S1. — Schematic diagram showing the transcription start sites (TSS) of KIAA0495, distribution of CpG dinucleotides (red lines), and amplicons of methylation-specific PCR (green box) and pyrosequencing (purple box). (TIFF 339 kb) [file 12943_2015_444_MOESM2_ESM.tif]
